# An investigation of cannabis use for insomnia in depression and anxiety in a naturalistic sample

**DOI:** 10.1186/s12888-022-03948-6

**Published:** 2022-04-28

**Authors:** Nirushi Kuhathasan, Luciano Minuzzi, James MacKillop, Benicio N. Frey

**Affiliations:** 1 Mood Disorders Program and Women’s Health Concerns Clinic, St. Joseph’s Healthcare Hamilton, 100 West 5th Street, Hamilton, ON L8N 3K7 Canada; 2 Michael G. DeGroote Centre for Medicinal Cannabis Research, McMaster University, 100 West 5th Street, Hamilton, ON L8N 3K7 Canada; 3 Department of Psychiatry and Behavioural Neurosciences, McMaster University, 100 West 5th Street, Hamilton, ON L8N 3K7 Canada; 4 Peter Boris Centre for Addictions Research, McMaster University/St. Joseph’s Healthcare Hamilton, 100 West 5th Street, Hamilton, ON L8N 3K7 Canada

**Keywords:** Medicinal cannabis, Insomnia, Depression, Anxiety, Symptom assessment, Linear mixed effects, Mobile health

## Abstract

**Background:**

Little is known about cannabis use for insomnia in individuals with depression, anxiety, and comorbid depression and anxiety. To develop a better understanding of distinct profiles of cannabis use for insomnia management, a retrospective cohort study was conducted on a large naturalistic sample.

**Methods:**

Data were collected using the medicinal cannabis tracking app, Strainprint®, which allows users to monitor and track cannabis use for therapeutic purposes. The current study examined users managing insomnia symptoms in depression (*n* = 100), anxiety (*n* = 463), and comorbid depression and anxiety (*n* = 114), for a total of 8476 recorded sessions. Inferential analyses used linear mixed effects modeling to examine self-perceived improvement across demographic variables and cannabis product variables.

**Results:**

Overall, cannabis was perceived to be efficacious across all groups, regardless of age and gender. Dried flower and oral oil were reported as the most used and most efficacious product forms. In the depression group, all strains were perceived to be efficacious and comparisons between strains revealed indica-dominant (*M*_diff_ = 1.81, 95% *CI* 1.26–2.36, *P*_adj_ < .001), indica hybrid (*M*_diff_ = 1.34, 95% *CI* 0.46–2.22, *P*_adj_ = .045), and sativa-dominant (*M*_diff_ = 1.83, 95% *CI* 0.68–2.99, *P*_adj_ = .028) strains were significantly more efficacious than CBD-dominant strains. In anxiety and comorbid conditions, all strain categories were perceived to be efficacious with no significant differences between strains.

**Conclusions:**

In terms of perceptions, individuals with depression, anxiety, and both conditions who use cannabis for insomnia report significant improvements in symptom severity after cannabis use. The current study highlights the need for placebo-controlled trials investigating symptom improvement and the safety of cannabinoids for sleep in individuals with mood and anxiety disorders.

**Supplementary Information:**

The online version contains supplementary material available at 10.1186/s12888-022-03948-6.

Disruptions in the sleep-wake cycle are a core component of the pathophysiology of mood and anxiety disorders. Insomnia is recognized as a common sleep disorder and may present itself as a comorbidity on both symptom and/or condition levels [1, 2]. In major depressive disorder (MDD), insomnia symptomology is reported by 80–90% of individuals, with poorer sleep associated with greater depressive symptom severity [3, 4]. Similarly, insomnia prevalence rates of 70–90% have been reported among individuals with anxiety disorders [3, 5], with co-occurring insomnia associated with increased risk of lifetime anxiety disorders [5]. Research on the relationship between sleep and mental illness have also reported insomnia as a post-treatment residual symptom in both mood and anxiety disorders [6–8]. Despite this, evidence-based strategies for treating insomnia in mood and anxiety disorders are limited, and first-line treatments for mood and anxiety disorders do little to manage insomnia symptoms in treatment-resistant individuals [9–11].

Interest in the use of cannabis products for therapeutic purposes has grown substantially with the recent legalization of the drug in several countries. A wide range of therapeutic advantages have been reported with cannabis use, with one commonly reported benefit being its use as a sleep aid [12]. Insomnia has been described as one of the primary reasons individuals seek medicinal cannabis [13], and approximately 1/4 of recreational users have reported that cannabis aids relaxation and sleep [14]. Similar findings have also been reported regarding cannabis use for depression and anxiety [15–17]. In fact, one study examining substitutions of medical cannabis for other pharmaceutical agents found that 71.8% of respondents reduced their use of anti-anxiety medications, 65.2% reduced their use of sleep medications, and 37.6% reduced their use of antidepressant medications with cannabis use [18]. A meta-analysis of medical cannabis use reported similar findings, with anxiety (50%) and depression (34%) among the top reasons for use [17]. Interestingly, in a retrospective study examining cannabis use for a variety of symptoms, cannabis use was also reported to provide the most relief in anxiety- and depression-related symptoms and insomnia presented the largest symptom relief score across all examined symptoms [19]. Despite this, several reviews have concluded that research on the benefits of cannabis for sleep are dominated by results from studies investigating other primary conditions, with sleep as a secondary outcome [20–22]. The present study aimed to investigate the use of cannabinoids for insomnia in individuals with depression, anxiety, and comorbid depression/anxiety and was conducted via app-based crowdsourced data from a large, naturalistic sample.

## Methods

A retrospective cohort study was conducted to examine use of cannabis products for insomnia symptoms in individuals with depression and/or anxiety. All data were anonymous and was obtained from the cannabis tracking app, Strainprint®. Using the app, subjects can record their conditions and symptoms, as well as a variety of cannabis usage variables. Of note, conditions and symptoms are subjectively determined and recorded by each individual. As such, it is possible that not all individuals meet full clinical diagnostic criteria for recorded conditions. At initial sign-up, all subjects provide consent to share their anonymized data for research purposes. Users cannot use the app or enter any data without agreeing to these terms. Once the agreement has been digitally accepted, users are prompted to enter basic demographic information. Prior to starting a session, subjects select relevant symptoms from a pre-populated dropdown list and are guided through instructions to rate the severity of their selected symptoms on a 0–10-point numeric rating scale (0-least severe; 10-very severe). Note that for the current study, we examined only insomnia symptoms in individuals with depression and/or anxiety. Next, subjects select the cannabis product they will use from a pre-populated list of products with lab-verified chemical ingredients for all medical cannabis products sold by licensed producers. Subjects then input additional information about the product and session, such as product form (flower, oil, capsule, edible, vape pen, concentrate), route of administration (vape, oil, smoke, edible, pill, tincture, spray, concentrate, dab bubble, dab portable, oral, topical, transdermal) and dose (drops, mg, ml, puffs). After an onset period defined by the selected route of administration (e.g., 20 min for smoke, 60 min for pill or edible), subjects are prompted with a push notification (occurs 8 h after initial ratings for insomnia symptoms) to complete a post-session rating of symptom severity on the same numeric scale.

The current study analyzed the data of participants who used cannabis to manage insomnia symptoms under conditions of depression and anxiety. Variables of interest were selected prior to data extraction, and Strainprint® subsequently provided all information stripped of any identifiers.

### Ethics approval

Ethics approval for this research was granted by the Hamilton Integrated Research Ethics Board (HiREB project #7162). The study was designed to be compliant with the Health and Information Protection Act, 2016 (HIPA).

### Study sample

The current study included sessions tracked from February 27, 2017 to February 28, 2020. All participants in this study experienced insomnia symptomology and used the Strainprint® app to monitor and track symptom severity pre- and post- cannabis use. Participants were stratified into groups based on their self-reported condition (i.e., only depression, only anxiety, comorbid depression and anxiety). In the depression condition, the sample consisted of 100 participants (*n* = 50 males; *n* = 50 females) tracking cannabis use across 976 recorded sessions. Participant ages ranged from 18 to 62 (M = 30.93, SD = 10.07). In the anxiety condition, 463 participants (*n* = 191 males; *n* = 269 females; *n* = 3 n/a) tracked usage across 4631 recorded sessions. Participant ages ranged from 18 to 71 (M = 31.43, SD = 8.91). Finally, in the comorbid condition, 114 participants (*n* = 60 males; *n* = 54 females) tracked usage across 2869 recorded sessions. Participant ages ranged from 18 to 62 (M = 33.98, SD = 10.70). Additional descriptive statistics on the samples are presented in Figs. S1, S2, S3.

### Statistical analysis

Descriptive analyses of the data were completed for each condition. Specifically, frequencies of categorical cannabis use variables (i.e., strain category and product form) were examined. The data were further stratified to investigate cannabis usage trends across age and gender. Inferential analyses for each condition examined self-perceived symptom improvement, which was calculated as the self-reported change in symptom severity between pre- and post- cannabis use.

To ensure validity of model results, plots were examined and determined not to violate the assumptions of normality and homoscedasticity (see Appendix for more detailed information on model diagnostics). Though regression analyses are commonly conducted for statistical modelling of this type, the current data reports multiple observations per subject, violating the assumption of independent observations in standard regression models. Standard regression analyses would not account for between-person variability in tracked sessions across subjects; therefore, linear mixed effects modeling (LMEM) was applied. LMEM corrects for non-independence in data and can estimate random intercepts and slopes to make accurate inferences. LMEM uses random effects to resolve between-subject variability and standard fixed effects to resolve within-subject variability, irrespective of differences in the frequency of observations reported per subject.

For the following analyses, LMEM was applied to predict changes in self-perceived symptom improvement across demographic variables (i.e., age and gender) and cannabis use variables (i.e., product form and strain category). Each condition was examined across all tracked sessions. Bonferroni corrections for multiple comparisons were applied to all results. Analyses were conducted using the statistical computing software, R.

## Results

### Strain categories and product forms

For each condition, descriptive analyses were performed across tracked sessions to examine the usage frequency of each strain category (i.e., sativa-dominant, sativa hybrid, indica-dominant, indica hybrid, balanced hybrid, cannabidiol [CBD]). Although CBD is not a plant strain, Strainprint® codes CBD-dominant products as a distinct category because of the varying amounts of THC (Δ ^9^-tetrahydrocannabinol)/CBD across different strains. Descriptive statistics examining strain categories for each condition are presented in Figs. S4, S5, S6.

The percentage frequency of each strain category for depression was examined across 976 sessions. Results indicate that CBD-dominant and indica-dominant strains were most used to manage insomnia symptoms in depression (Fig. S4). Strain category usage frequency was examined across 4631 sessions in the anxiety condition (Fig. S5) and across 2869 sessions in the comorbid condition (Fig. S6). In both anxiety and comorbid conditions, indica-dominant and indica hybrid strains were most used to manage insomnia. Notably, across all conditions, sativa-dominant and sativa hybrid strains were least used.

Descriptive analyses of cannabis product forms (i.e., flower, oil, other) for the management of insomnia are stratified by age and gender and frequencies (Tables S1-S2, S3). Cannabis was most often used in the form of dried flower across most conditions, except in individuals between 35 and 44 years of age with depression, and in individuals 45+ years of age with anxiety or comorbid conditions, who used oil more often.

### Linear mixed effects model predictions of self-perceived symptom improvement

Figures S7, S8, S9 present bar graphs of insomnia symptom severity ratings for each condition before and after cannabis use. Fig. S7 examines pre-medication (*M* = 6.76, *SD* = 1.90) and post-medication (*M* = 3.24, *SD* = 2.87) insomnia symptom severity across tracked sessions for the depression condition (*n* = 100 users across 976 sessions). Fig. S8 examines pre-medication (*M* = 7.24, *SD* = 1.86) and post-medication (*M* = 3.61, *SD* = 2.55) symptom severity across tracked sessions for the anxiety condition (*n* = 463 users across 4631 sessions). Finally, Fig. S9 examines pre-medication (*M* = 7.10, *SD* = 2.01) and post-medication (*M* = 2.73, *SD* = 2.26) symptom severity across tracked sessions in the comorbid condition (*n* = 114 users across 2869 sessions).

#### Depression

Cannabis was perceived as significantly efficacious (*P*_adj_ < 0.01) for most age groups in the depression condition (Table 1). Interestingly, cannabis was not perceived as efficacious for the 45+ age group. There were no significant differences in self-perceived symptom improvement found between age groups (Table S4).

**Table 1:** Depression: Age X Self-Perceived Insomnia Symptom Improvement

	Estimate	Std. Error	df	t value	***p*** value	Cohen’s D [95% CI]
18–24	2.755	0.495	188.2	5.569	3.504e-07***	0.41 [0.26, 0.55]
25–34	2.669	0.409	93.1	6.524	1.409e-08***	0.68 [0.45, 0.90]
35–44	3.087	0.726	73.7	4.251	2.462e-04***	0.50 [0.25, 0.74]
45+	2.339	1.113	116.9	2.102	0.151	0.19 [0.01, 0.38]

Table 1: *N*_sessions_ = 976; *N*_subjects_ = 100. Self-perceived insomnia symptom improvement by age group in depression. The self-perceived symptom improvement was tested using linear mixed modeling (beta coefficient was not standardized). ***p < .001

Self-perceived symptom improvement was also examined across various product forms and was significantly efficacious in the form of a flower or oil (*P*_adj_ < 0.01) (Table S5). Product forms with an insufficient number of observations to warrant inclusion in principal analyses (i.e., those making up < 10% of the data) were collapsed into one distinct group. Product forms of this group, consisting of capsules, edibles, vape pens, concentrates, and tinctures, were not found to be efficacious. Additional comparisons between product forms found no significant differences (Table S6).

Finally, the self-perceived symptom improvement of cannabis strain categories was examined (Table 2). All strains were perceived by the participants be efficacious improving insomnia symptoms in the depression group (*P*_adj_ < 0.01). When self-perceived symptom improvement was compared between strain categories, indica-dominant (*M*_diff_ = 1.81, 95% *CI* 1.26–2.36, *P*_adj_ < .001), indica hybrid (*M*_diff_ = 1.34, 95% *CI* 0.46–2.22, *P*_adj_ = .045), and sativa-dominant (*M*_diff_ = 1.83, 95% *CI* 0.68–2.99, *P*_adj_ = .028) strains were found to be significantly more efficacious than CBD-dominant strains (Table 3).

**Table 2:** Depression: Strain Category X Self-Perceived Insomnia Symptom Improvement

	Estimate	Std. Error	df	t value	p value	Cohen’s D [95% CI]
Balanced Hybrid	2.483	0.442	266.3	5.618	< 1.32e-15 ***	0.34 [0.22, 0.47]
CBD-dominant	1.498	0.410	183.8	3.657	< 1.32e-15 ***	0.27 [0.12, 0.42]
Indica-dominant	3.306	0.374	139.2	8.844	< 1.32e-15 ***	0.75 [0.56, 0.94]
Indica Hybrid	2.836	0.405	193.7	6.998	< 1.32e-15 ***	0.50 [0.35, 0.65]
Sativa-dominant	3.329	0.579	427.0	5.745	< 1.32e-15 ***	0.28 [0.18, 0.37]
Sativa Hybrid	2.699	0.702	504.2	3.843	< 1.32e-15 ***	0.17 [0.08, 0.26]

Table 2: *N*_sessions_ = 976; *N*_subjects_ = 100. Self-perceived insomnia symptom improvement by strain category in depression. The self-perceived symptom improvement was tested used linear mixed modeling (beta coefficient was not standardized). ***p < .001

**Table 3:** Depression: Strain Category Self-Perceived Insomnia Symptom Improvement Comparisons

	Estimate	Std. Error	df	t value	p value	Cohen’s D [95% CI]
Balanced Hybrid – CBD-dominant	0.985	0.426	966.3	2.312	0.315	0.07 [0.01, 0.14]
Balanced Hybrid – Indica-dominant	− 0.823	0.389	964.5	−2.116	0.519	− 0.07 [− 0.13, 0.00]
Balanced Hybrid – Indica Hybrid	− 0.353	0.446	953.9	− 0.792	1.000	−0.03 [− 0.09, 0.04]
Balanced Hybrid – Sativa-dominant	− 0.846	0.616	949.0	−1.374	1.000	−0.04 [− 0.11, 0.02]
Balanced Hybrid – Sativa Hybrid	−0.216	0.746	841.8	−0.289	1.000	−0.01 [− 0.08, 0.06]
CBD-dominant – Indica-dominant	−1.808	0.279	947.7	−6.478	2.240e-09***	−0.21 [− 0.27, − 0.15]
CBD-dominant – Indica Hybrid	−1.338	0.450	851.6	−2.974	0.045**	−0.10 [− 0.17, − 0.03]
CBD-dominant – Sativa-dominant	−1.831	0.588	931.1	−3.112	0.028**	−0.10 [− 0.17, − 0.04]
CBD-dominant – Sativa Hybrid	−1.201	0.744	780.8	−1.614	1.000	−0.06 [− 0.13, 0.01]
Indica-dominant – Indica Hybrid	0.470	0.413	836.0	1.139	1.000	0.04 [−0.03, 0.11]
Indica-dominant – Sativa-dominant	−0.023	0.550	932.7	−0.041	1.000	−0.001 [− 0.07, 0.06]
Indica-dominant – Sativa Hybrid	0.607	0.721	775.6	0.842	1.000	0.03 [−0.04, 0.10]
Indica Hybrid – Sativa-dominant	−0.493	0.614	876.2	−0.802	1.000	−0.03 [− 0.09, 0.04]
Indica Hybrid – Sativa Hybrid	0.137	0.704	845.0	0.195	1.000	0.007 [−0.06, 0.07]
Sativa-dominant – Sativa Hybrid	0.630	0.845	832.5	0.746	1.000	0.03 [−0.04, 0.09]

Table 3: *N*_sessions_ = 976; *N*_subjects_ = 100. Self-perceived insomnia symptom improvement comparisons between strain categories in depression. The self-perceived symptom improvement was tested used linear mixed modeling (beta coefficient was not standardized). ***p < .001., **p < .01

#### Anxiety

When examined as a function of age, cannabis was efficacious across all age groups in the anxiety condition (*P*_adj_ < 0.01) (Table S7), and comparisons between age groups found cannabis to be more efficacious in the 35–44 age group over the 25–34 age group (*M*_diff_ = 1.07, 95% *CI* 0.46–1.67, *P*_adj_ = .0004) (Table 4).

**Table 4:** Anxiety: Age Group Self-Perceived Insomnia Symptom Improvement Comparisons

	Estimate	Std. Error	df	t value	p value	Cohen’s D [95% CI]
[18–24] – [25–34]	0.669	0.305	586.7	2.192	0.173	0.18 [0.02, 0.34]
[18–24] – [35–44]	−0.397	0.362	451.8	−1.098	1.000	−0.10 [− 0.29, 0.08]
[18–24] – [45+]	0.208	0.495	413.9	0.421	1.000	0.04 [− 0.15, 0.23]
[25–34] – [35–44]	−1.066	0.307	434.8	−3.474	0.004***	−0.33 [− 0.52,-0.14]
[25–34] – [45+]	−0.460	0.458	395.0	−1.006	1.000	−0.10 [− 0.30, 0.10]
[35–44] – [45+]	0.606	0.489	394.8	1.238	1.000	0.12 [−0.07, 0.32]

Table 4: *N*_sessions_ = 4631; *N*_subjects_ = 463. Self-perceived insomnia symptom improvement comparisons between age groups in anxiety. The self-perceived symptom improvement was tested used linear mixed modeling (beta coefficient was not standardized). ***p < .001

When self-perceived symptom improvement was examined by product, all forms (Table S8) and strain categories (Table S9) were found to be efficacious for anxiety (*P*_adj_ < 0.01). Comparisons between product forms (Table S10) and strain categories (Table S11) found no significant differences.

#### Comorbid depression and anxiety

Finally, cannabis was perceived to be efficacious across all age groups in the comorbid group (*P*_adj_ < 0.01) (Table S12).

When self-perceived symptom improvement was examined by product form, all forms were found to be efficacious (Table S13); however, comparisons between groups found cannabis in the form of an oil to be slightly more efficacious than flower (*M*_diff_ = 0.51, 95% *CI* 0.14–0.87, *P*_adj_ = .019) and other forms (*M*_diff_ = 1.32, 95% *CI* 0.35–2.30, *P*_adj_ = .024) (Table 5). An examination of strain categories found all strains to be efficacious (*P*_adj_ < 0.01) (Table S14) with no significant differences between strains (Table S15).

**Table 5:** Comorbid: Product Form Self-Perceived Insomnia Symptom Improvement Comparisons

	Estimate	Std. Error	df	t value	p value	Cohen’s D [95% CI]
Flower – Oil	−0.505	0.185	2734.1	−2.731	0.019**	−0.05 [− 0.09, − 0.01]
Flower – Other	0.818	0.478	1951.1	1.711	0.262	0.04 [−0.01, 0.08]
Oil – Other	1.323	0.498	2007.7	2.658	0.024**	0.06 [0.02, 0.10]

Table 5: N_sessions_ = 2869; *N*_subjects_ = 114. Self-perceived insomnia symptom improvement comparisons between product forms in comorbid condition. The self-perceived symptom improvement was tested used linear mixed modeling (beta coefficient was not standardized). **p < .01

## Discussion

The present study was conducted to investigate cannabis use profiles and self-perceived symptom improvement for insomnia in individuals with depression, anxiety, and comorbid anxiety and depression through crowdsourced health data. Self-reported scores before and after cannabis use indicate a significant self-perceived benefit with the use of cannabinoids for insomnia. These findings are consistent with preliminary results from clinical trials, suggesting that cannabis may be a future option for insomnia management [23].

In our study, all cannabis strains were perceived to improve insomnia by individuals with depression, anxiety, and comorbid depression and anxiety; however, in individuals with depression, CBD-dominant products were felt to be less efficacious than indica-dominant, indica hybrid, and sativa-dominant strains to improve insomnia. This could suggest that individuals with depression have a distinct response profile to CBD for insomnia, and/or CBD might exert anxiolytic effects in individuals with anxiety and comorbid depression/anxiety, which, in turn, may improve sleep. Interestingly, previous studies have suggested that insomnia may have independent relationships with depression and anxiety [5] and it is possible that this finding is a result of distinct pathways for the relationships between depression and insomnia, and between anxiety and insomnia, respectively. Previous research suggests that insomnia could have etiologically distinct directional associations with anxiety versus depression, supporting the hypothesis that the nature of the relationship between insomnia and mental disorders may be different depending on the comorbid condition [5]. These results are in line with the varying responses to cannabis for insomnia in anxiety versus depression in our study.

Notably, our study also compared the self-perceived efficacy of cannabis for insomnia symptoms across all age ranges. The current literature on the influence of age in cannabis and sleep outcomes is relatively scarce. However, the function of the endocannabinoid system in circadian rhythms has been well-established and emerging evidence has highlighted its potential modulating role in the regulation of sleep within the context of aging [24–26]. There are also reports of differences in the pharmacokinetics of cannabis with increased age, which may potentially influence how the drug is absorbed in older adults [24, 26–28]. As younger adults have faster basal metabolisms, it has been theorized that differences in cannabis-related effects across age groups may be explained by the unique biological effects of aging [24, 29]. Furthermore, previous studies examining sleep across age has consistently reported decreases in sleep quality, shorter sleep times, and more fragmented sleep with older adults [26, 30, 31]. Some studies have also reported age-related variability in the presentation of symptoms of major depressive disorder, with older adults reporting more sleep-related depressive symptomology, including problems sleeping during the night and more early morning awakenings [32]. Though cannabis was perceived efficacious across most age groups in our study, this was not true for older adults in the depressive group.

Despite the potential benefits of cannabinoids for insomnia, research in the field lacks placebo-controlled trials that assess self-perceived symptom improvement alongside risks and harms. Though some studies suggest that administration of THC and THC-derivatives, alone or in combination with CBD may improve sleep outcomes, very few clinical studies have objectively investigated the efficacy of cannabis for sleep using validated measures and sleep as a primary outcome [20–22]. Large placebo-controlled trials using both objective and subjective measures of sleep parameters are warranted. Additionally, given the highly comorbid nature of depression, anxiety, and sleep disorders, placebo-controlled trials investigating the use of cannabis for the management of insomnia in these populations are encouraged.

### Limitations

The present study has several limitations. First, reported conditions and symptoms were determined subjectively by individuals; as a result, it is unclear whether all individuals meet full diagnostic criteria for these conditions. Importantly, the lack of an objective measure of insomnia in our study is a main limitation. Although insomnia severity in the present study is measured on a 0–10-point scale, insomnia may manifest itself in different ways that were not captured with this app. Furthermore, Strainprint® collects a very specific set of information from each individual. Any additional data that may influence symptom improvement outcomes (e.g., medical history, concurrent medications, etc.) were not able to be assessed. The present study may also involve some sampling bias. As Strainprint® is largely marketed to cannabis users, resulting samples may underrepresent individuals who find cannabis to be ineffective and overrepresent those who benefit from its use. Moreover, information on potential side effects is lacking; therefore, any data regarding negative subject experiences from cannabis use are inaccessible. Additionally, the current study examined strain categories, though differences between categories remain largely controversial [33–35]. Among consumers, different strains are often associated with various perceived effects [36]; however, many researchers maintain that any perceived effects are a result of other components of cannabis (ex. terpenes) which are not typically reported to consumers [33, 34]. As such, it is possible that perceived effects reported in this study are a result of interactions between various cannabis components rather than individual strains specifically. Nonetheless, in the absence of robust RCTs, investigations of perceived effects of strain categories in naturalistic settings can improve understanding of consumer purchasing decisions [37], as well as inform future trial designs.

Despite its limitations, this study is strengthened by its large, naturalistic sample. Individuals were also prompted to record cannabis use in their daily environments, maximizing ecological validity of the study. As such, large mobile health studies of this sort are considerably more convenient and provide real-time information. Although in real life many people report using cannabis use for depression, anxiety and sleep, this area of research is still relatively scarce. As such, results from the naturalistic study can provide a better understanding of cannabis usage profiles for insomnia, while providing valuable information for future trials designed to evaluate efficacy and safety of cannabis for therapeutic purposes.

## Conclusion

This naturalistic investigation of cannabis use for insomnia suggests that individuals with depression, anxiety, and comorbid depression and anxiety perceive benefits from using cannabis for sleep, although the extent to which this reflects pharmacological efficacy versus response expectancies (i.e., placebo effects) cannot be ascertained. In addition, compared to other cannabis strains, CBD-dominant products may be less helpful for sleep, specifically in individuals with depression. The current study highlights the need for placebo-controlled trials investigating the efficacy and safety of cannabinoids for sleep in individuals with mood and anxiety disorders.

## Supplementary Information

**Additional file 1.**

## Data Availability

The data that support the findings of this study are available from Strainprint® but restrictions apply to the availability of these data, which were used under license for the current study, and so are not publicly available. Data are however available from the authors upon reasonable request and with permission of Strainprint®.
