# Supplementary material for: An investigation of cannabis use for insomnia in depression and anxiety in a naturalistic sample
Source: BMC Psychiatry. 2022 Apr 28;22:303. doi: 10.1186/s12888-022-03948-6 (PMC9052466; doi:10.1186/s12888-022-03948-6)
Supplement: Supplementary file 1 — Additional file 1. [file 12888_2022_3948_MOESM1_ESM.docx]

**Supplementary Figures**

**DEPRESSION: Demographics**

FIGURE S1: Descriptive information on demographics for depression condition.

**ANXIETY: Demographics**

FIGURE S2: Descriptive information on demographics for anxiety condition.

**COMORBID: Demographics**

FIGURE S3: Descriptive information on demographics for comorbid condition.

**DEPRESSION: Strain Categories**

FIGURE S4: Descriptive information on frequency of strain categories used in depression across 976 tracked sessions.

**ANXIETY: Strain Categories**

FIGURE S5: Descriptive information on frequency of strain categories used in anxiety across 4631 tracked sessions

**COMORBID: Strain Categories**

FIGURE S6: Descriptive information on frequency of strain categories used in comorbid condition across 2869 tracked sessions

**DEPRESSION: Pre- & Post-Medication Insomnia Symptom Severity**

**
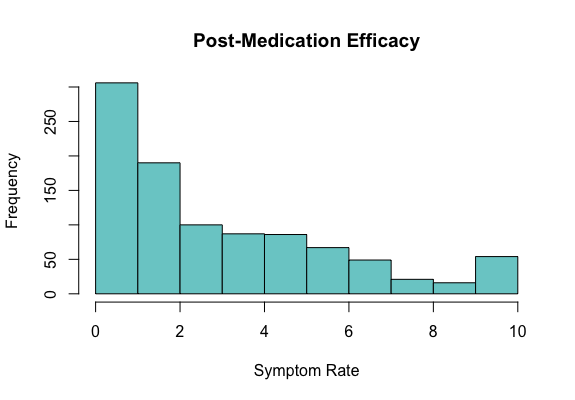

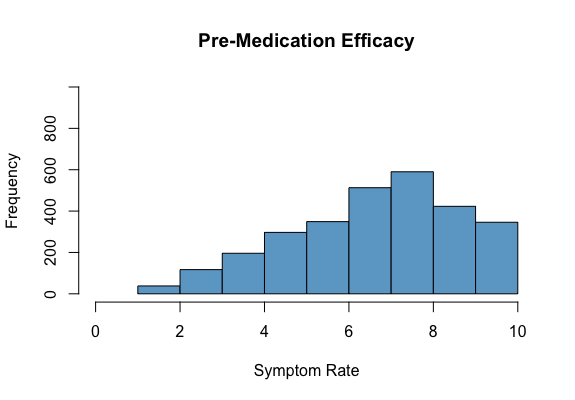
**

FIGURE S7: Pre- (*M*=6.76, *SD*=1.90) and post-medication (*M*=3.24, *SD*=2.87) insomnia symptom severity in depression across 976 tracked sessions (n=100 users).

**ANXIETY: Pre- & Post-Medication Insomnia Symptom Severity**

**
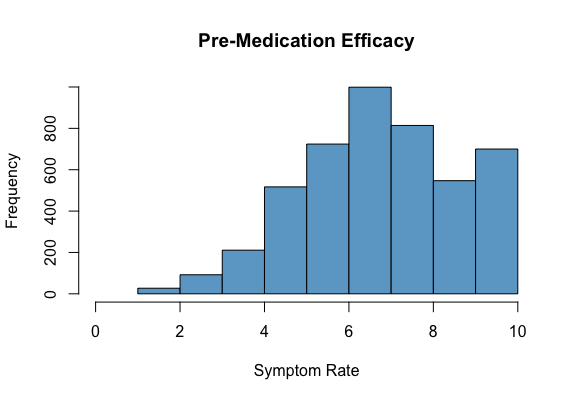

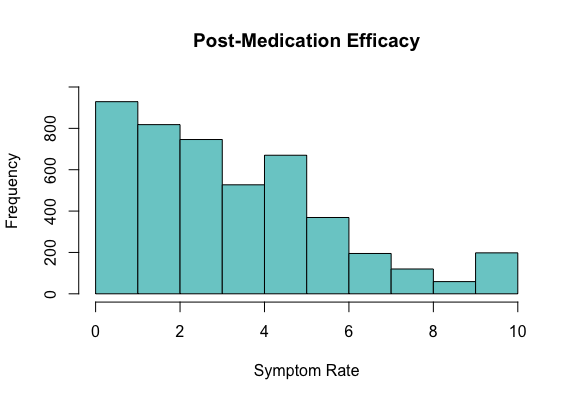
**

FIGURE S8: Pre-(*M*=7.24, *SD*=1.86) and post-medication (*M*=3.61, *SD*=2.55) insomnia symptom severity in anxiety across 4631 tracked sessions (n=463 users).

**COMORBID: Pre- & Post-Medication Insomnia Symptom Severity**

**
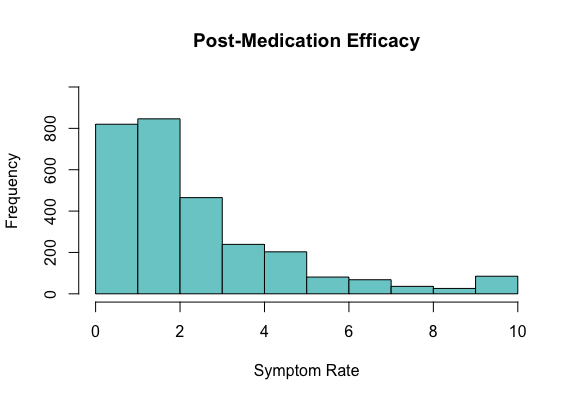

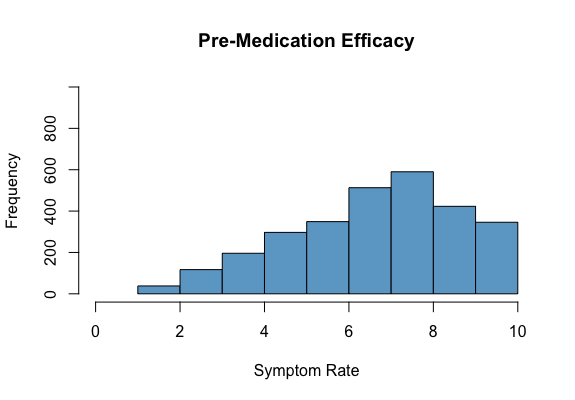
**

FIGURE S9: Pre-(*M*=7.10, *SD*=2.01) and post-medication (*M*=2.73, *SD*=2.26) insomnia symptom severity in comorbid condition across 2869 tracked sessions (n=114 users).

**Supplementary Tables**

**DEPRESSION: Frequency of Cannabis Product Forms**

| Product Form | By Gender | Female | Male | By Age | 18-24 | 25-34 | 35-44 | 45+ |
| --- | --- | --- | --- | --- | --- | --- | --- | --- |
| Flower |  | 285 | 488 |  | 81 | 520 | 127 | 45 |
| Oil |  | 171 | 22 |  | 2 | 29 | 154 | 8 |
| Other |  | 2 | 8 |  | 0 | 10 | 0 | 0 |

TABLE S1: Frequency of cannabis product forms used in depression across 976 sessions between genders and age groups.

**ANXIETY: Frequency of Cannabis Product Forms**

| Product Form | By Gender | Female | Male | By Age | 18-24 | 25-34 | 35-44 | 45+ |
| --- | --- | --- | --- | --- | --- | --- | --- | --- |
| Flower |  | 1643 | 1548 |  | 563 | 1603 | 878 | 146 |
| Oil |  | 243 | 1128 |  | 369 | 214 | 283 | 503 |
| Other |  | 49 | 20 |  | 7 | 15 | 39 | 7 |

TABLE S2: Frequency of cannabis product forms used in anxiety across 4631 sessions between genders and age groups.

**COMORBID: Frequency of Cannabis Product Forms**

| Product Form | By Gender | Female | Male | By Age | 18-24 | 25-34 | 35-44 | 45+ |
| --- | --- | --- | --- | --- | --- | --- | --- | --- |
| Flower |  | 1245 | 653 |  | 270 | 582 | 988 | 58 |
| Oil |  | 346 | 566 |  | 61 | 132 | 268 | 451 |
| Other |  | 25 | 34 |  | 4 | 22 | 24 | 9 |

TABLE S3: Frequency of cannabis product forms used in comorbid condition across 2869 sessions between genders and age groups.

**DEPRESSION: Age Group Self-Perceived Insomnia Symptom Improvement Comparisons**

|  | **Estimate** | **Std. Error** | **df** | **t value** | **p value** | **Cohen’s D**  [95% CI] |
| --- | --- | --- | --- | --- | --- | --- |
| [18-24] – [25-34] | 0.086 | 0.494 | 507.2 | 0.173 | 1.000 | 0.02  [-0.16, 0.19] |
| [18-24] – [35-44] | -0.332 | 0.879 | 95.4 | -0.378 | 1.000 | -0.08  [-0.48, 0.32] |
| [18-24] – [45+] | 0.416 | 1.218 | 125.6 | 0.341 | 1.000 | 0.06  [-0.29, 0.41] |
| [25-34] – [35-44] | -0.418 | 0.834 | 77.8 | -0.501 | 1.000 | -0.11  [-0.56, 0.33] |
| [25-34] – [45+] | 0.330 | 1.186 | 113.6 | 0.278 | 1.000 | 0.05  [-0.32, 0.42] |
| [35-44] – [45+] | 0.748 | 1.329 | 100.8 | 0.563 | 1.000 | 0.11  [-0.28, 0.50] |

TABLE S4: *N*_sessions_ = 976; *N*_subjects_ = 100. Self-perceived insomnia symptom improvement comparisons between age groups in depression. The self-perceived symptom improvement was tested used linear mixed modeling (beta coefficient was not standardized).

**DEPRESSION: Product Form X Self-Perceived Insomnia Symptom Improvement**

|  | **Estimate** | **Std. Error** | **df** | **t value** | **p value** | **Cohen’s D**  [95% CI] |
| --- | --- | --- | --- | --- | --- | --- |
| Flower | 2.808 | 0.318 | 85.0 | 8.829 | 3.579e-13*** | 0.96  [0.70, 1.21] |
| Oil | 2.239 | 0.476 | 305.0 | 4.707 | 1.148e-05*** | 0.27  [0.16, 0.38] |
| Other | 2.281 | 1.396 | 729.7 | 1.633 | 0.308 | 0.06  [-0.01, 0.13] |

TABLE S5: *N*_sessions_ = 976; *N*_subjects_ = 100. Self-perceived insomnia symptom improvement by product form in depression. The self-perceived symptom improvement was tested used linear mixed modeling (beta coefficient was not standardized). ****p* < .001.

**DEPRESSION: Product Form Self-Perceived Insomnia Symptom Improvement Comparisons**

|  | **Estimate** | **Std. Error** | **df** | **t value** | **p value** | **Cohen’s D**  [95% CI] |
| --- | --- | --- | --- | --- | --- | --- |
| Flower – Oil | 0.568 | 0.395 | 972.9 | 1.440 | 0.451 | 0.05 [-0.02, .11] |
| Flower – Other | 0.527 | 1.380 | 834.8 | 0.382 | 1.000 | 0.01 [-0.05, 0.08] |
| Oil – Other | -0.041 | 1.431 | 842.8 | -0.029 | 1.000 | -0.001 [-0.07, 0.07] |

TABLE S6: *N*_sessions_ = 976; *N*_subjects_ = 100. Self-perceived insomnia symptom improvement comparisons between product forms in depression. The self-perceived symptom improvement was tested used linear mixed modeling (beta coefficient was not standardized).

**ANXIETY: Age X Self-Perceived Insomnia Symptom Improvement**

|  | **Estimate** | **Std. Error** | **df** | **t value** | **p value** | **Cohen’s D**  [95% CI] |
| --- | --- | --- | --- | --- | --- | --- |
| 18-24 | 3.216 | 0.259 | 515.7 | 12.406 | < 8.8e-16*** | 0.55 [0.45, 0.64] |
| 25-34 | 2.547 | 0.179 | 471.0 | 14.243 | < 8.8e-16*** | 0.66 [0.56, 0.76] |
| 35-44 | 3.613 | 0.253 | 391.7 | 14.288 | < 8.8e-16*** | 0.72 [0.61, 0.83] |
| 45+ | 3.008 | 0.421 | 382.9 | 7.137 | 1.924e-11*** | 0.36 [0.26, 0.47] |

TABLE S7: *N*_sessions_ = 4631; *N*_subjects_ = 463. Self-perceived insomnia symptom improvement by age in anxiety. The self-perceived symptom improvement was tested used linear mixed modeling (beta coefficient was not standardized). ****p* < .001.

**ANXIETY: Product Form X Self-Perceived Insomnia Symptom Improvement**

|  | **Estimate** | **Std. Error** | **df** | **t value** | **p value** | **Cohen’s D**  [95% CI] |
| --- | --- | --- | --- | --- | --- | --- |
| Flower | 2.956 | 0.129 | 440.7 | 22.840 | < 6.6e-16*** | 1.09 [0.97, 1.21] |
| Oil | 3.006 | 0.173 | 1026.7 | 17.363 | < 6.6e-16*** | 0.54 [0.48, 061] |
| Other | 2.992 | 0.499 | 3207.3 | 5.992 | 6.924e-09*** | 0.11 [0.07, 0.14] |

TABLE S8: *N*_sessions_ = 4631; *N*_subjects_ = 463. Self-perceived insomnia symptom improvement by product form in anxiety. The self-perceived symptom improvement was tested used linear mixed modeling (beta coefficient was not standardized). ****p* < .001.

**ANXIETY: Strain Category X Self-Perceived Insomnia Symptom Improvement**

|  | **Estimate** | **Std. Error** | **df** | **t value** | **p value** | **Cohen’s D**  [95% CI] |
| --- | --- | --- | --- | --- | --- | --- |
| Balanced Hybrid | 2.854 | 0.168 | 1044.7 | 17.017 | < 1.32e-15*** | 0.53 [0.46, 0.59] |
| CBD-dominant | 2.882 | 0.166 | 989.7 | 17.347 | < 1.32e-15*** | 0.55 [0.48, 0.62] |
| Indica-dominant | 3.005 | 0.149 | 722.4 | 20.162 | < 1.32e-15*** | 0.75 [0.67, 0.83] |
| Indica Hybrid | 3.002 | 0.150 | 744.2 | 19.959 | < 1.32e-15*** | 0.73 [0.65, 0.81] |
| Sativa-dominant | 3.472 | 0.235 | 2625.0 | 14.743 | < 1.32e-15*** | 0.29 [0.25, 0.33] |
| Sativa Hybrid | 2.678 | 0.291 | 2985.8 | 9.210 | < 1.32e-15*** | 0.17 [0.13, 0.20] |

TABLE S9: *N*_sessions_ = 4631; *N*_subjects_ = 463. Self-perceived insomnia symptom improvement by strain category in anxiety. The self-perceived symptom improvement was tested used linear mixed modeling (beta coefficient was not standardized). ****p* < .001.

**ANXIETY: Product Form Self-Perceived Insomnia Symptom Improvement Comparisons**

|  | **Estimate** | **Std. Error** | **df** | **t value** | **p value** | **Cohen’s D**  [95% CI] |
| --- | --- | --- | --- | --- | --- | --- |
| Flower – Oil | -0.050 | 0.145 | 4375.8 | -0.346 | 1.000 | -0.005 [-0.03, 0.02] |
| Flower – Other | -0.036 | 0.495 | 3682.5 | -0.073 | 1.000 | -0.001 [-0.03, 0.03] |
| Oil – Other | 0.014 | 0.503 | 3773.3 | 0.028 | 1.000 | 0.0004 [-0.03, 0.03] |

TABLE S10: *N*_sessions_ = 4631; *N*_subjects_ = 463. Self-perceived insomnia symptom improvement comparisons between product forms in anxiety. The self-perceived symptom improvement was tested used linear mixed modeling (beta coefficient was not standardized).

**ANXIETY: Strain Category Self-Perceived Insomnia Symptom Improvement Comparisons**

|  | **Estimate** | **Std. Error** | **df** | **t value** | **p value** | **Cohen’s D**  [95% CI] |
| --- | --- | --- | --- | --- | --- | --- |
| Balanced Hybrid – CBD-dominant | -0.028 | 0.165 | 4625.0 | -0.171 | 1.000 | -0.003 [-0.03, 0.03] |
| Balanced Hybrid – Indica-dominant | -0.151 | 0.153 | 4622.6 | -0.990 | 1.000 | -0.01 [-0.04, 0.01] |
| Balanced Hybrid – Indica Hybrid | -0.148 | 0.153 | 4625.0 | -0.970 | 1.000 | -0.01 [-0.04, 0.01] |
| Balanced Hybrid – Sativa-dominant | -0.618 | 0.238 | 4616.6 | -2.593 | 0.142 | -0.04 [-0.07, -0.01] |
| Balanced Hybrid – Sativa Hybrid | 0.176 | 0.296 | 4511.7 | 0.594 | 1.000 | 0.009 [-0.02, 0.04] |
| CBD-dominant – Indica-dominant | -0.123 | 0.154 | 4577.3 | -0.799 | 1.000 | -0.01 [-0.04, 0.02] |
| CBD-dominant – Indica Hybrid | -0.120 | 0.153 | 4602.4 | -0.783 | 1.000 | -0.01 [-0.04, 0.02] |
| CBD-dominant – Sativa-dominant | -0.590 | 0.239 | 4624.6 | -2.466 | 0.206 | -0.04 [-0.07, -0.01] |
| CBD-dominant – Sativa Hybrid | 0.204 | 0.298 | 4470.5 | 0.685 | 1.000 | 0.01 [-0.02, 0.04] |
| Indica-dominant – Indica Hybrid | 0.003 | 0.133 | 4620.6 | 0.026 | 1.000 | 0.0004 [-0.03, 0.03] |
| Indica-dominant – Sativa-dominant | -0.466 | 0.222 | 4608.1 | -2.098 | 0.540 | -0.03 [-0.06, 0.00] |
| Indica-dominant – Sativa Hybrid | 0.328 | 0.290 | 4471.0 | 1.13 | 1.000 | 0.02 [-0.01, 0.05] |
| Indica Hybrid – Sativa-dominant | -0.470 | 0.225 | 4609.7 | -2.089 | 0.552 | -0.03 [-0.06, 0.00] |
| Indica Hybrid – Sativa Hybrid | 0.324 | 0.287 | 4509.0 | 1.131 | 1.000 | 0.02 [-0.01, 0.05] |
| Sativa-dominant – Sativa Hybrid | 0.794 | 0.334 | 4600.4 | 2.378 | 0.262 | 0.04 [0.01, 0.06] |

TABLE S11: *N*_sessions_ = 4631; *N*_subjects_ = 463. Self-perceived insomnia symptom improvement comparisons between strain categories in anxiety. The self-perceived symptom improvement was tested used linear mixed modeling (beta coefficient was not standardized).

**COMORBID: Age X Self-Perceived Insomnia Symptom Improvement**

|  | **Estimate** | **Std. Error** | **df** | **t value** | **p value** | **Cohen’s D**  [95% CI] |
| --- | --- | --- | --- | --- | --- | --- |
| 18-24 | 3.577 | 0.507 | 118.8 | 7.051 | 5.052e-10*** | 0.65 [0.45, 0.84] |
| 25-34 | 2.881 | 0.435 | 126.9 | 6.618 | 3.697e-09*** | 0.59 [0.40, 0.77] |
| 35-44 | 3.386 | 0.457 | 120.0 | 7.417 | 7.492e-11*** | 0.68 [0.48, 0.87] |
| 45+ | 3.276 | 0.664 | 112.8 | 4.936 | 1.110e-05*** | 0.46 [0.27, 0.66] |

TABLE S12: N_sessions_ = 2869; *N*_subjects_ = 114. Self-perceived insomnia symptom improvement by age in comorbid condition. The self-perceived symptom improvement was tested used linear mixed modeling (beta coefficient was not standardized). ****p* < .001.

**COMORBID: Product Form X Self-Perceived Insomnia Symptom Improvement**

|  | **Estimate** | **Std. Error** | **df** | **t value** | **p value** | **Cohen’s D**  [95% CI] |
| --- | --- | --- | --- | --- | --- | --- |
| Flower | 3.189 | 0.258 | 118.5 | 12.374 | < 6.6e-16*** | 1.14 [0.90, 1.37] |
| Oil | 3.694 | 0.289 | 176.2 | 12.803 | < 6.6e-16*** | 0.96 [0.78, 1.14] |
| Other | 2.371 | 0.513 | 724.3 | 4.622 | 1.351e-05*** | 0.17 [0.10, 0.25] |

TABLE S13: *N*_sessions_ = 2869; *N*_subjects_ = 114. Self-perceived insomnia symptom improvement by product form in comorbid condition. The self-perceived symptom improvement was tested used linear mixed modeling (beta coefficient was not standardized). ****p* < .001.

**COMORBID: Strain Category X Self-Perceived Insomnia Symptom Improvement**

|  | **Estimate** | **Std. Error** | **df** | **t value** | **p value** | **Cohen’s D**  [95% CI] |
| --- | --- | --- | --- | --- | --- | --- |
| Balanced Hybrid | 3.471 | 0.292 | 183.6 | 11.884 | < 1.32e-15 *** | 0.88 [0.71, 1.05] |
| CBD-dominant | 3.386 | 0.292 | 183.5 | 11.600 | < 1.32e-15 *** | 0.86 [0.69, 1.02] |
| Indica-dominant | 3.244 | 0.264 | 124.5 | 12.309 | < 1.32e-15 *** | 1.10 [0.88, 1.32] |
| Indica Hybrid | 3.011 | 0.280 | 157.2 | 10.738 | < 1.32e-15 *** | 0.86 [0.67, 1.04] |
| Sativa-dominant | 3.629 | 0.385 | 503.5 | 9.421 | < 1.32e-15 *** | 0.42 [0.33, 0.51] |
| Sativa Hybrid | 2.760 | 0.359 | 409.0 | 7.691 | 6.588e-13 *** | 0.38 [0.28, 0.48] |

TABLE S14: *N*_sessions_ = 2869; *N*_subjects_ = 114. Self-perceived insomnia symptom improvement by strain category in comorbid condition. The self-perceived symptom improvement was tested used linear mixed modeling (beta coefficient was not standardized). ****p* < .001.

**COMORBID: Strain Category Self-Perceived Insomnia Symptom Improvement Comparisons**

|  | **Estimate** | **Std. Error** | **df** | **t value** | **p value** | **Cohen’s D**  [95% CI] |
| --- | --- | --- | --- | --- | --- | --- |
| Balanced Hybrid – CBD-dominant | 0.085 | 0.228 | 2854.6 | 0.372 | 1.000 | 0.007 [-0.03, 0.04] |
| Balanced Hybrid – Indica-dominant | 0.227 | 0.184 | 2861.9 | 1.233 | 1.000 | 0.02 [-0.01, 0.06] |
| Balanced Hybrid – Indica Hybrid | 0.460 | 0.194 | 2860.8 | 2.366 | 0.270 | 0.04 [0.01, 0.08] |
| Balanced Hybrid – Sativa-dominant | -0.158 | 0.327 | 2857.8 | -0.481 | 1.000 | -0.009 [-0.05, 0.03] |
| Balanced Hybrid – Sativa Hybrid | 0.711 | 0.282 | 2807.9 | 2.518 | 0.179 | 0.05 [0.01, 0.08] |
| CBD-dominant – Indica-dominant | 0.142 | 0.177 | 2860.0 | 0.803 | 1.000 | 0.02 [-0.02, 0.05] |
| CBD-dominant – Indica Hybrid | 0.375 | 0.216 | 2849.8 | 1.734 | 1.000 | 0.03 [0.00, 0.07] |
| CBD-dominant – Sativa-dominant | -0.243 | 0.332 | 2860.2 | -0.731 | 1.000 | -0.01 [-0.05, 0.02] |
| CBD-dominant – Sativa Hybrid | 0.626 | 0.301 | 2841.1 | 2.080 | 0.564 | 0.04 [0.00, 0.08] |
| Indica-dominant – Indica Hybrid | 0.233 | 0.160 | 2862.8 | 1.454 | 1.000 | 0.03 [-0.01, 0.06] |
| Indica-dominant – Sativa-dominant | -0.385 | 0.311 | 2857.5 | -1.238 | 1.000 | -0.02 [-0.06, 0.01] |
| Indica-dominant – Sativa Hybrid | 0.484 | 0.274 | 2816.6 | 1.770 | 1.000 | 0.03 [0.00 0.07] |
| Indica Hybrid – Sativa-dominant | -0.618 | 0.320 | 2858.4 | -1.932 | 0.802 | -0.04 [-0.07, 0.00] |
| Indica Hybrid – Sativa Hybrid | 0.251 | 0.278 | 2814.0 | 0.901 | 1.000 | 0.02 [-0.02, 0.05] |
| Sativa-dominant – Sativa Hybrid | 0.869 | 0.357 | 2823.8 | 2.436 | 0.224 | 0.05 [0.01, 0.08] |

TABLE S15: *N*_sessions_ = 2869; *N*_subjects_ = 114. Self-perceived insomnia symptom improvement comparisons between strain categories in comorbid condition. The self-perceived symptom improvement was tested used linear mixed modeling (beta coefficient was not standardized).

**Appendix**

**Model Diagnostics**

| **Model** | **Skew of Residuals** | **Skew of Random Effects** |
| --- | --- | --- |
| **Depression** | | |
| Age | -0.78 | -0.27 |
| Product Form | -0.79 | 0.03 |
| Strain Category | -0.72 | 0.00 |
| **Anxiety** | | |
| Age | -0.68 | -0.10 |
| Product Form | -0.68 | -0.09 |
| Strain Category | -0.67 | 0.22 |
| **Comorbid** | | |
| Age | -1.32 | -0.72 |
| Product Form | -1.31 | -0.68 |
| Strain Category | -1.28 | -0.71 |

Table A1: Skewness values of residual and random effects for each model.


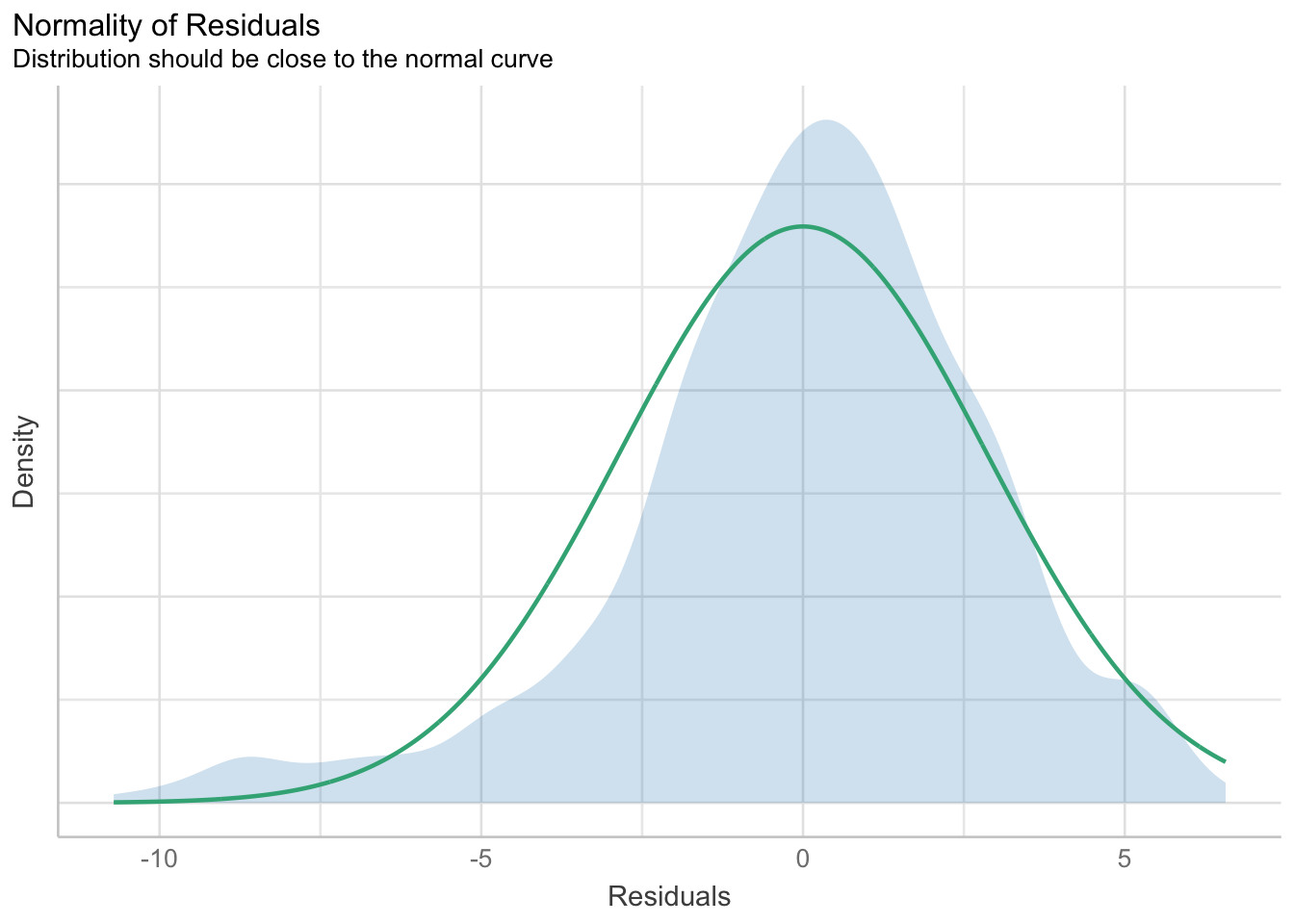


Figure A1: Density Plot of Residuals for Age Efficacy (Depression) Model


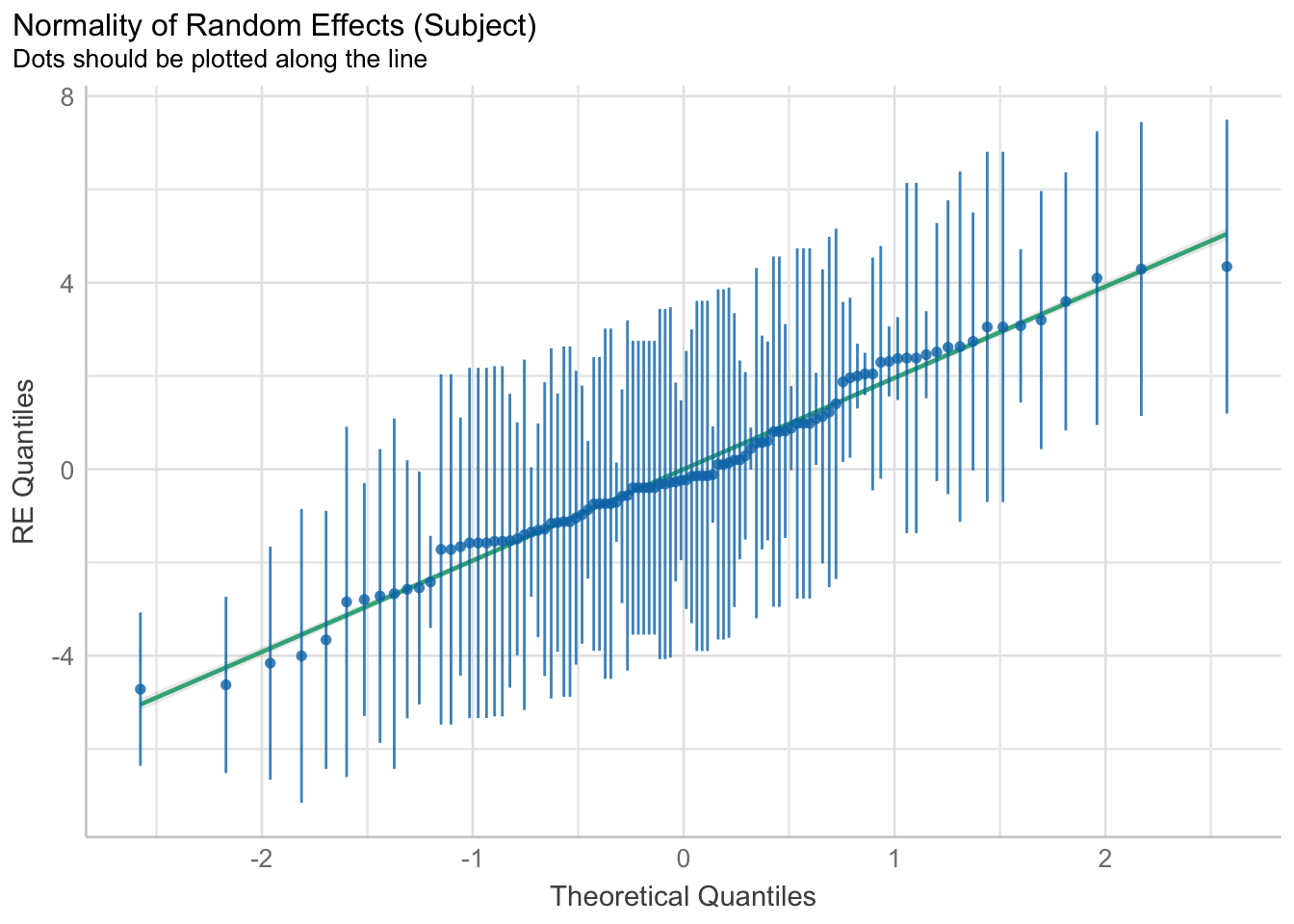


Figure A2: Q-Q Plot of Random Effects for Age Efficacy (Depression) Model


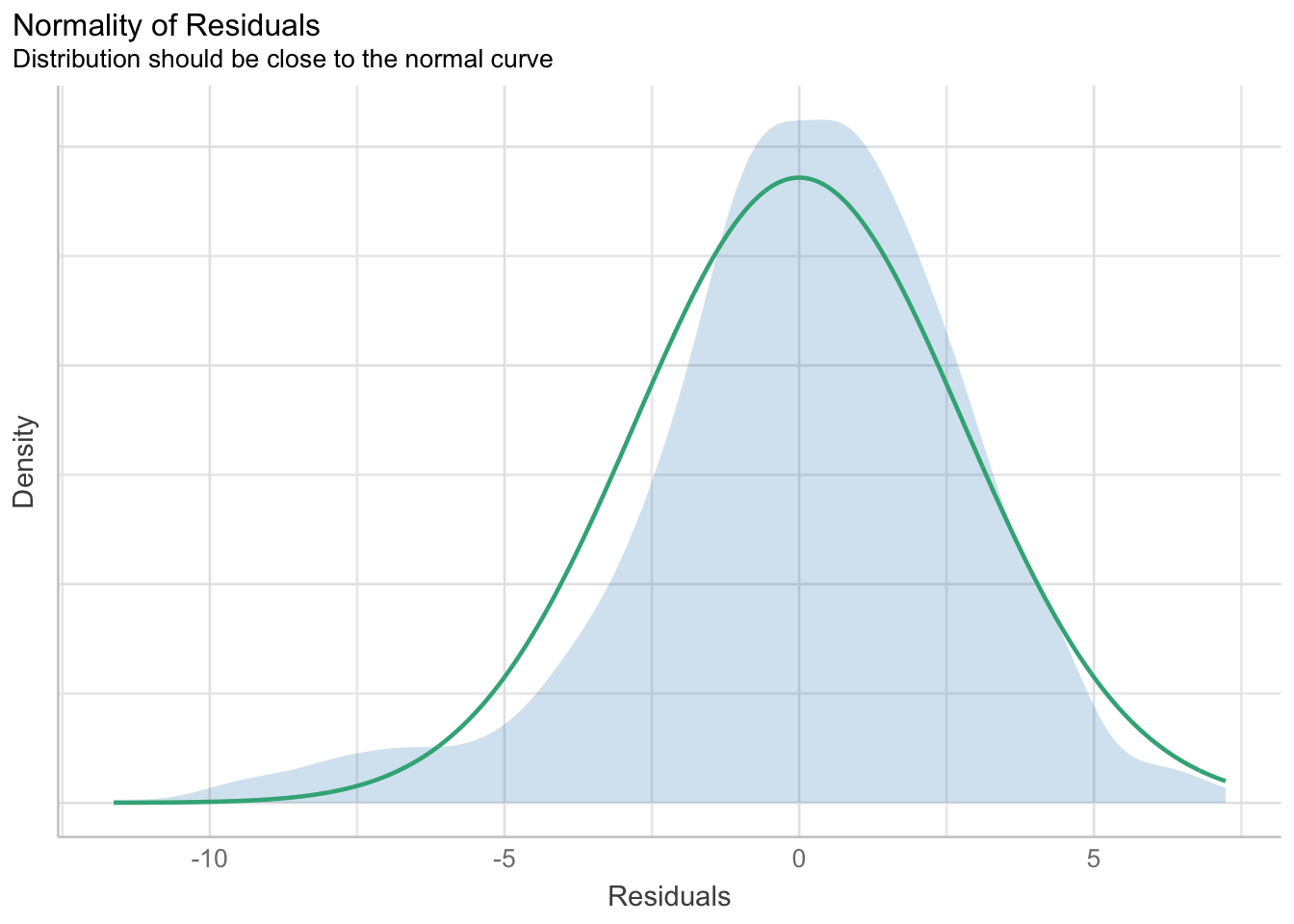


Figure A3: Density Plot of Residuals for Strain Category Efficacy (Depression) Model


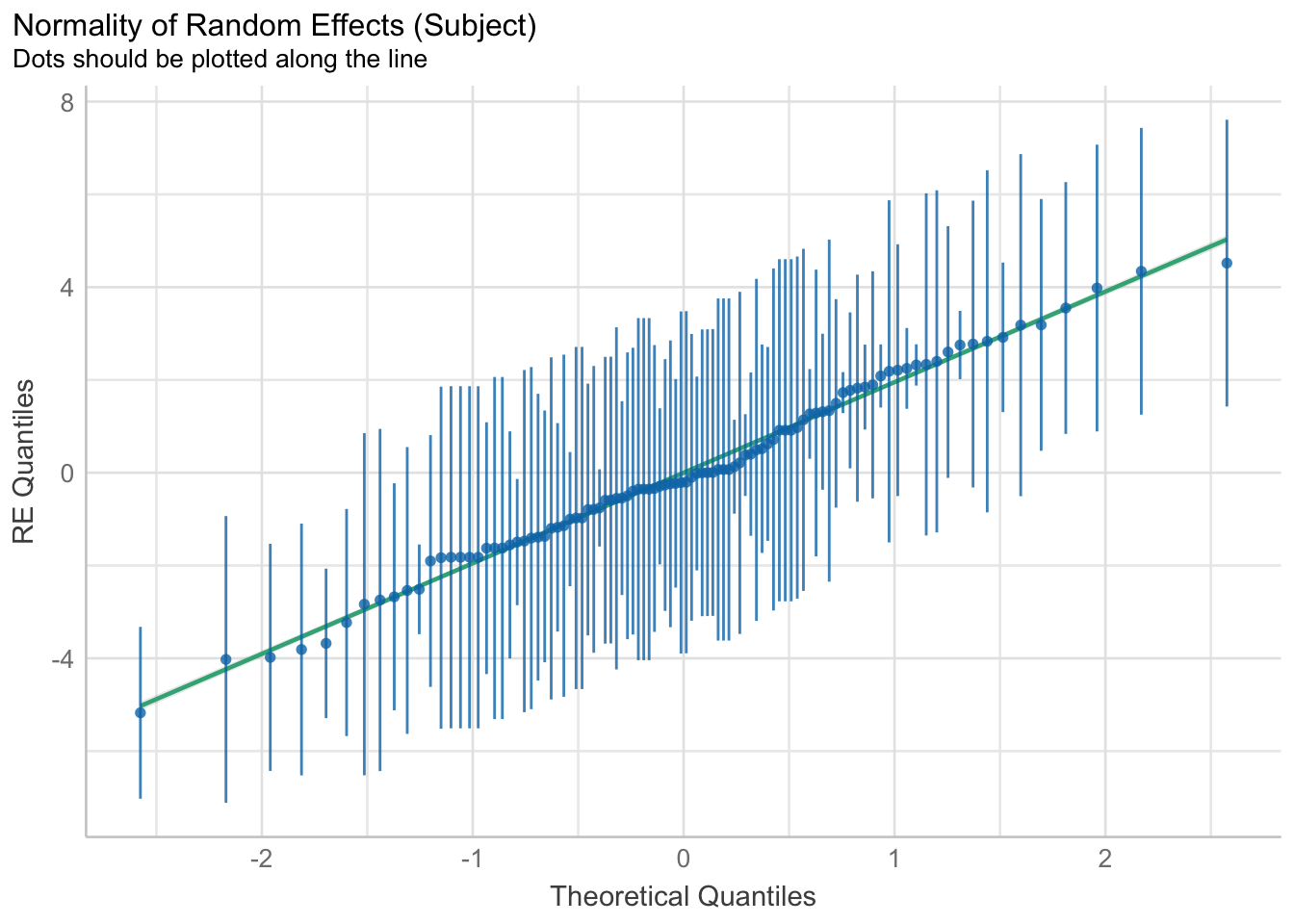


Figure A4: Q-Q Plot of Random Effects for Strain Category Efficacy (Depression) Model


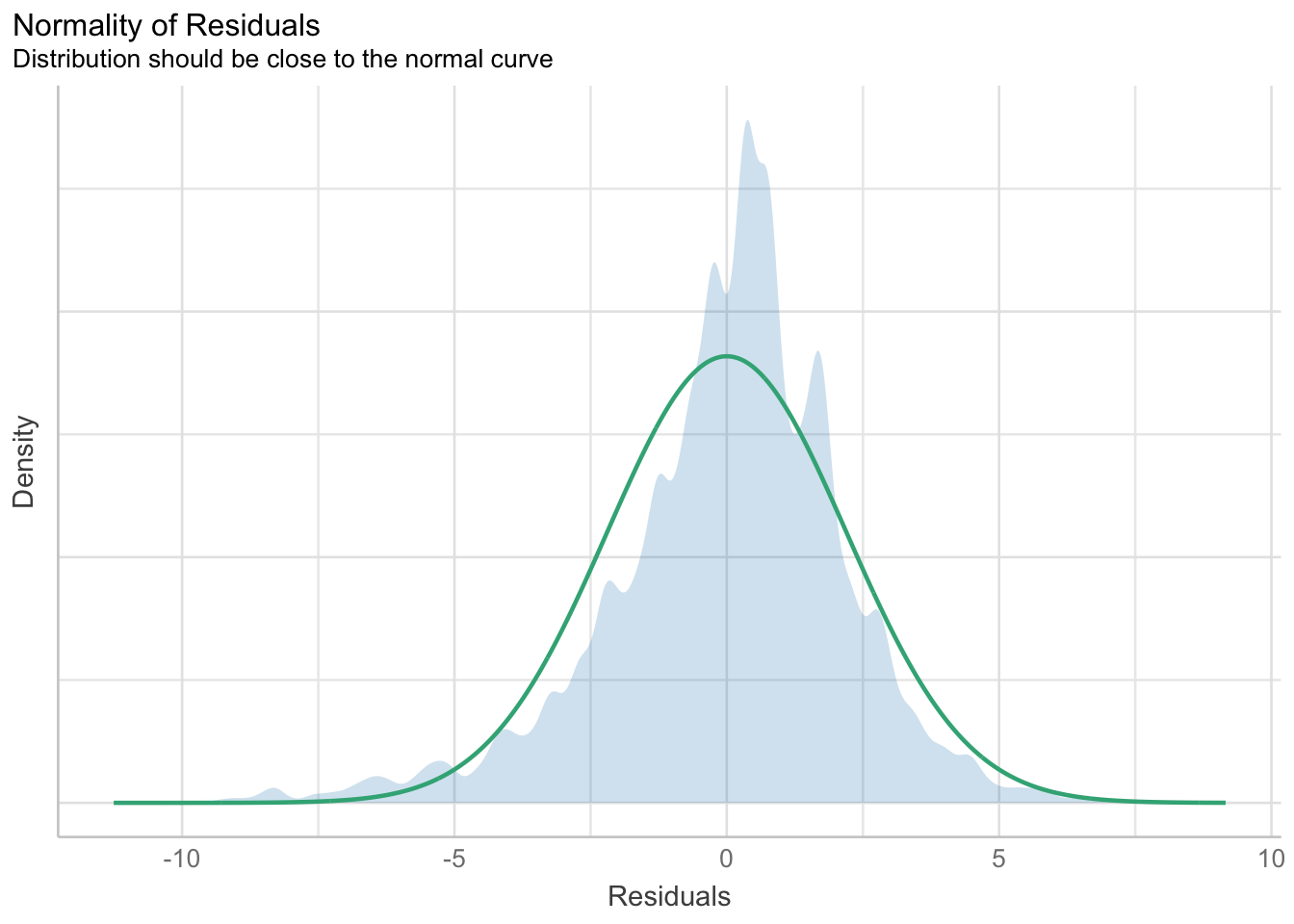


Figure A5: Density Plot of Residuals for Age Efficacy (Anxiety) Model


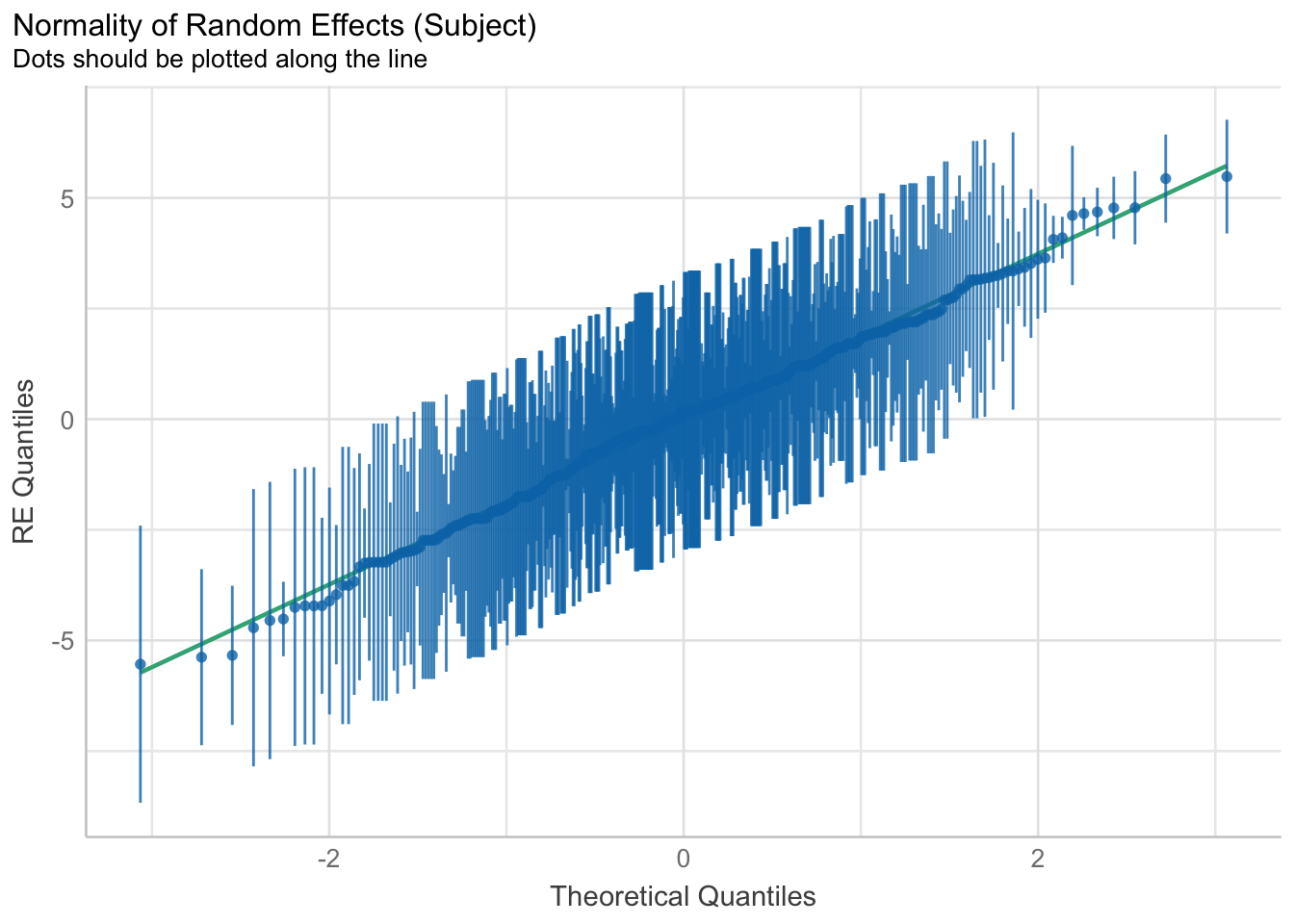


Figure A6: Q-Q Plot of Random Effects for Age Efficacy (Anxiety) Model


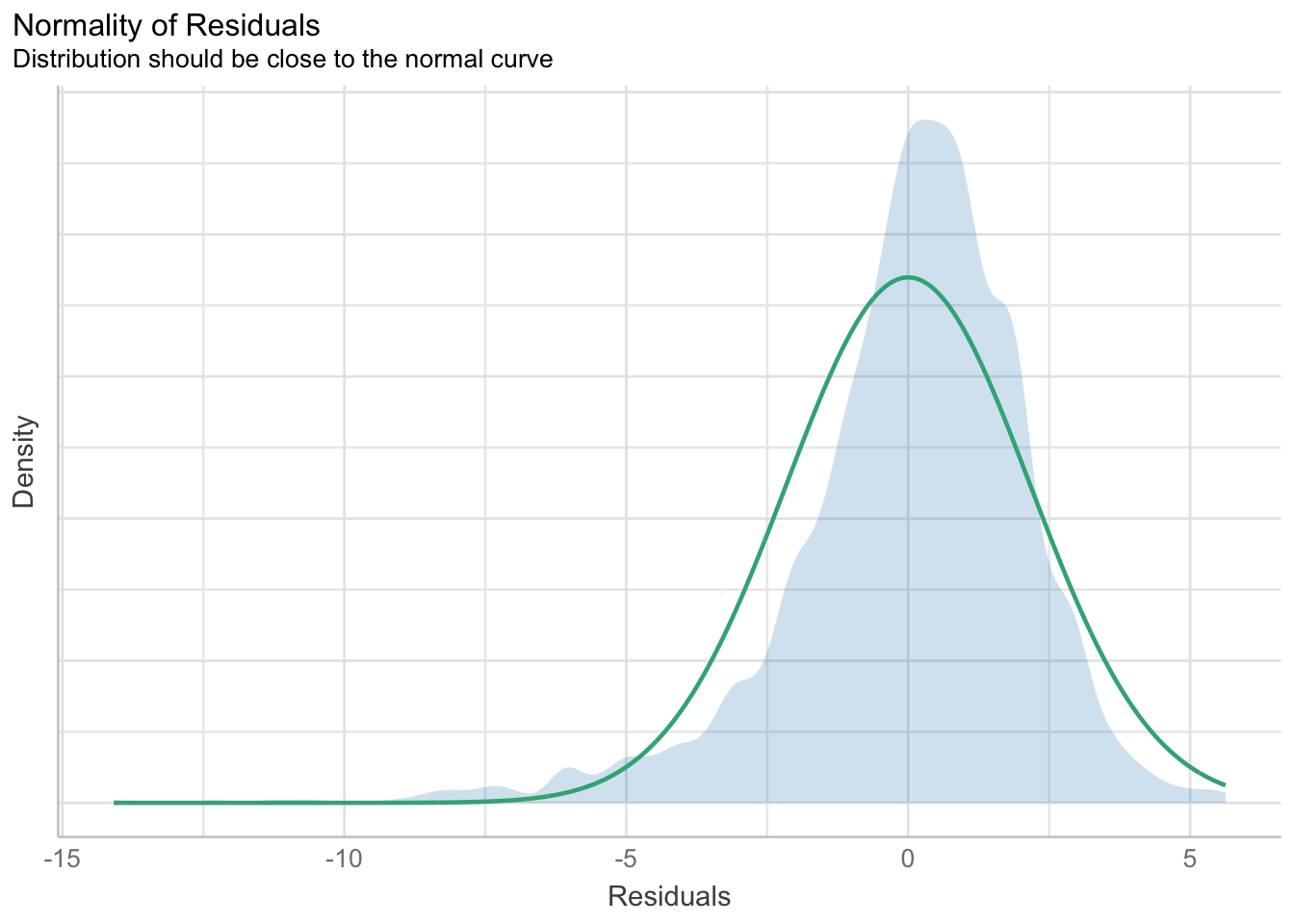


Figure A7: Density Plot of Residuals for Product Form (Comorbid) Model


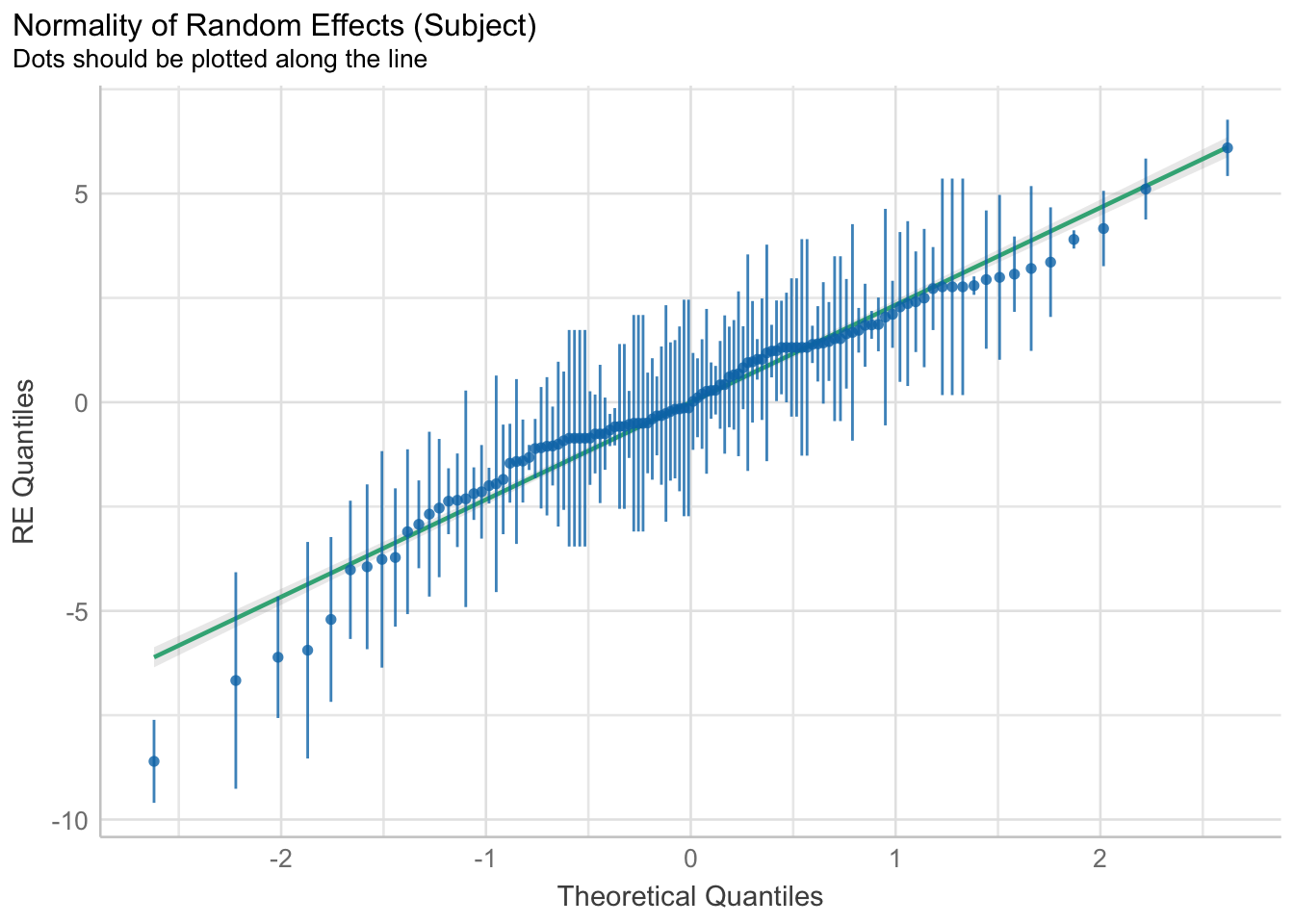


Figure A8: Q-Q Plot of Random Effects for Product Form (Comorbid) Model
